# Physiological and Pathological Inflammation Induced by Antibodies and Pentraxins

**DOI:** 10.3390/cells10051175

**Published:** 2021-05-12

**Authors:** Chiara Elisabeth Geyer, Lynn Mes, Melissa Newling, Jeroen den Dunnen, Willianne Hoepel

**Affiliations:** 1 Amsterdam Rheumatology and Immunology Center, Department of Rheumatology and Clinical Immunology, Amsterdam UMC, Meibergdreef 9, 1105 AZ Amsterdam, The Netherlands; c.e.geyer@amsterdamumc.nl (C.E.G.); l.mes1@amsterdamumc.nl (L.M.); melissanewling@hotmail.com (M.N.); j.w.hoepel@amsterdamumc.nl (W.H.); 2 Department of Experimental Immunology, Amsterdam UMC, Amsterdam Infection and Immunity Institute, University of Amsterdam, Meibergdreef 9, 1105 AZ Amsterdam, The Netherlands; 3 Department of Medical Microbiology, Amsterdam UMC, University of Amsterdam, Meibergdreef 9, 1105 AZ Amsterdam, The Netherlands

**Keywords:** macrophages, Fc receptors, antibodies, pentraxins, IgG, IgA

## Abstract

Macrophages play a key role in induction of inflammatory responses. These inflammatory responses are mostly considered to be instigated by activation of pattern recognition receptors (PRRs) or cytokine receptors. However, recently it has become clear that also antibodies and pentraxins, which can both activate Fc receptors (FcRs), induce very powerful inflammatory responses by macrophages that can even be an order of magnitude greater than PRRs. While the physiological function of this antibody-dependent inflammation (ADI) is to counteract infections, undesired activation or over-activation of this mechanism will lead to pathology, as observed in a variety of disorders, including viral infections such as COVID-19, chronic inflammatory disorders such as Crohn’s disease, and autoimmune diseases such as rheumatoid arthritis. In this review we discuss how physiological ADI provides host defense by inducing pathogen-specific immunity, and how erroneous activation of this mechanism leads to pathology. Moreover, we will provide an overview of the currently known signaling and metabolic pathways that underlie ADI, and how these can be targeted to counteract pathological inflammation.

## 1. Introduction

Macrophages play a crucial role in counteracting infections with pathogens. Upon recognition of invading pathogens, macrophages induce inflammatory responses that do not only activate cells in the local tissues, but also shape adaptive immune responses by subsequent activation of T cells. Since macrophages are strong immune modulators, unwanted or excessive inflammation can lead to immune pathology. Initially, these inflammatory responses by macrophages were considered to be predominantly induced by so-called pattern recognition receptors (PRRs), which recognize conserved foreign structures on pathogens known as pathogen-associated molecular patterns (PAMPs). In addition to these PAMPs, PRRs can also recognize danger-associated molecular patterns (DAMPs), which are endogenous structures released upon tissue damage or cell death [1,2]. To be able to detect different types of invading pathogens, PRRs are expressed at different compartments within innate immune cells. Toll-like receptors (TLRs) are the best characterized PRRs, consisting of ten family members that are located both intracellularly (TLR3,7,8,9) and extracellularly (TLR1,2,4,5,6,10). TLRs sense a broad range of pathogen-derived structures, e.g., TLR3, TLR4, and TLR9, which, respectively recognize double-stranded RNA, LPS, or unmethylated CpG DNA originating from bacteria or viruses. Examples of other PRR families are the C-type lectin receptors (CLR), RIG-I-like receptors, and NOD-like receptors [1,3,4].

Importantly, in addition to PRRs, in recent years also antibodies and pentraxins have been identified to play a key role in the induction of inflammation by macrophages. In this review, we will provide an overview of how antibodies and pentraxins induce inflammatory responses by macrophages, and we will discuss how this physiological mechanism can lead to pathology in various different disorders.

### Antibodies and Fc Receptors

**Figure 1:**
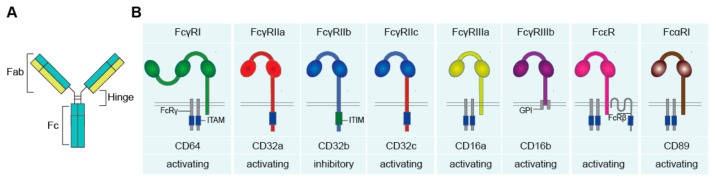
The human Fc receptors for IgG (FcγR), IgA (FcαR), and IgE (FcεR). (**A**) Schematic figure of an antibody, comprising of two Fab and one Fc region, connected via the hinge region. (**B**) Fc receptors recognize antibodies upon binding of the Fc tail to the extracellular Ig-like domains. Upon activation, FcRs can induce activating and inhibitory downstream signaling, mediated via ITAM and ITIM motives. Abbreviations: Fab, fragment antigen binding; Fc, fragment crystallizable; Ig, immunoglobulins; ITAM, immunoreceptor tyrosine-based activating motif; FcRγ, FcR gamma chain; ITIM, immunoreceptor tyrosine-based inhibitory motif; GPI, glycosylphosphatidylinositol; FcRβ, FcR beta chain.

Antibodies, also referred to as immunoglobulins (Ig), are a crucial component of the human immune system. Antibodies comprise a general “Y-shape” structure of two fragment antigen binding (Fab) regions, which are connected with the fragment crystallizable (Fc) region via the hinge region (Figure 1A).

The Fab region binds antigens, whereas the Fc region can interact with Fc receptors (FcRs) on the cell surface of macrophages [5,6]. There are five main classes of antibodies, known as isotypes, i.e., IgG, IgA, IgM, IgD, and IgE. IgG and IgA can be further subdivided into four (IgG1–4) and two subclasses (IgA1 and 2), respectively. IgG is the most abundant isotype present in human serum [6], while IgA is the most abundantly produced antibody in the human body and is predominantly present at mucosal sites.

Antibodies have a broad range of functions. Neutralization of pathogens is one of the main functions of antibodies. Invading pathogens are bound by antibodies, which are abundantly present throughout the body, with only a few excluded tissues such as the central nervous system. Neutralization by binding of antibodies to pathogens can prevent infection and concomitant pathology [5]. In addition to neutralization, antibodies can also induce several other antibody-mediated effector functions. The most well-known effector functions are antibody-dependent cellular phagocytosis (ADCP), antibody-dependent cellular cytotoxicity (ADCC) and complement-dependent cytotoxicity (CDC) [6,7]. These effector functions of antibodies have been extensively reviewed by others [5,6,7,8,9,10,11]. In this review we will focus on a more recently identified effector function of antibodies, i.e., their capacity to induce inflammation, which we will here refer to as antibody-dependent inflammation (ADI).

ADI is a powerful effector function whereby activation of FcγR strongly up- or down-regulates cytokine production induced by PRRs. Similar to other antibody effector functions such as the induction of phagocytosis and cytotoxicity, ADI is triggered by the interaction of antibodies with FcRs. There is a broad variety of FcRs, which differ in affinity for different types of antibodies, downstream signaling, and expression on different cell types (Figure 1B). In general, each antibody isotype interacts with a specific class of FcRs.; IgG binds to Fc gamma receptors (FcγRs), IgA binds to Fc alpha receptors (FcαRs), and IgE to Fc epsilon receptors (FcεRs). FcγRs, FcαRs, and FcεRs recognize the Fc tail of antibodies through their extracellular Ig-like domains [1]. Importantly, FcRs differ in their affinity for antibodies. FcRs with a high affinity can bind monomeric antibodies, while FcRs with a lower affinity require the formation of antibody immune complexes, which occurs for instance after recognition of opsonized pathogens [5].

FcγRs (recognizing IgG) can be classified into three family members, i.e., FcγRI, FcγRII, and FcγRIII [11]. FcγRI (CD64) is a high affinity receptor in humans that expresses three extracellular Ig-like domains. FcγRI has no intracellular signaling tail, but can associate with the FcR gamma chain (FcRγ), located in the cytoplasm, which bears an immunoreceptor tyrosine-based activation motif (ITAM), required for further downstream signaling [1,5,12].

FcγRII (CD32) expresses two extracellular Ig-like domains and can be subdivided into FcγRIIa, FcγRIIb, and FcγRIIc. FcγRIIa is a low affinity receptor that signals via its own cytoplasmic tail, which carries an ITAM. FcγRIIb is generally considered to be an “inhibitory receptor” and is often simultaneously expressed with activating FcγRs, which provides negative feedback to prevent over-activation. FcγRIIb counteracts activating signals via activation of the immunoreceptor tyrosine-based inhibitory motif (ITIM) expressed on the cytoplasmic tail [11,13]. FcγRIIc is only expressed in approximately 20% of the population that carries the *FCGR2C*-open reading frame (ORF) polymorphism, which makes it possible to express this receptor. FcγRIIc consist of the extracellular binding domain of FcγRIIb and the intracellular domain of FcγRIIa [13,14].

FcγRIII (CD16) consists of two subfamilies: FcγRIIIa and FcγRIIIb, which both express two extracellular Ig-like domains. FcγRIIIa signals via the cytoplasmic FcRγ that expresses an ITAM. FcγRIIIb however has no intracellular domain but expresses a glycosylphosphatidylinositol (GPI) anchor, which intracellular signaling function is still not disclosed [10]. 

FcαRI (CD89), which has two extracellular Ig-like domains, consists of only one family member and has a low affinity for IgA. FcαRI has no intracellular signaling domain, but signals also via FcRγ, which bears an ITAM motif [1,15].

Besides the classical Ig-like domain containing FcRs there are also other receptors that bind antibodies, such as the neonatal FcR (FcRn) and cytosolic tripartite motif (TRIM)21 [1]. FcRn has an important function in the transportation of IgG across the membrane, which is crucial to deliver humoral immunity into newborns, but also for recycling and thereby prolonging the half-life of IgGs in humans [16,17,18]. TRIM21 recognizes the Fc part of antibodies and can induce intracellular immune signaling, but also antibody degradation by linking the recognized antibody to the ubiquitin proteasome system [19]. 

## 2. Physiological Immune Activation: Host Defense against Pathogens

The main physiological function of ADI is to induce an inflammatory response that provides protective immunity upon infection with pathogens such as bacteria and viruses. This is mediated through the production of key pro-inflammatory cytokines and chemokines, such as TNF, IL-1β, IL-6, IL-8, and IL-23. This inflammatory response by macrophages and other myeloid immune cells does not only induce local immune activation, but also shapes subsequent differentiation of CD4 and CD8 T cells [20,21]. Thus far, ADI has mainly been described to be induced by humans [22], most likely because it is dependent on receptors that are restrictively expressed in animals, such as FcγRIIa and FcαRI. While FcRs are also expressed on B cells [23], T cells [24], neutrophils [25], and even non-immune cells such as endothelium [26], and epithelium [27,28], ADI seems most pronounced for myeloid antigen-presenting cells (APCs) such as macrophages, monocytes, dendritic cells (DC), Langerhans cells (LCs), Kupffer cells, and microglia [20,21,29,30,31,32,33,34,35].

**Figure 2:**
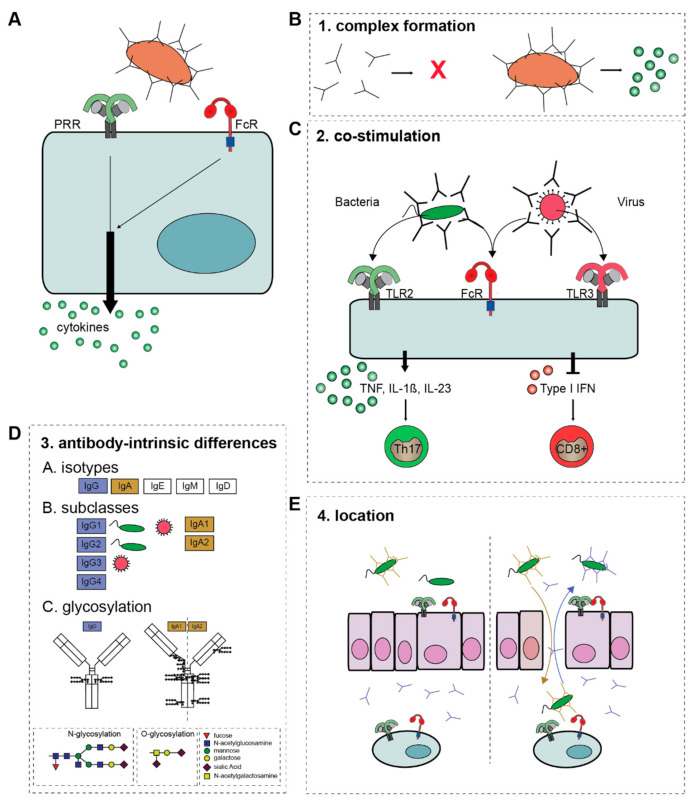
The four criteria of ADI activation. (**A**) FcR-PRR cross-talk modulates PRR-induced cytokine production. The overall outcome of ADI depends on four criteria. (**B**) 1. Antibody-complex formation, (**C**) 2. The co-stimulus that FcRs collaborate with, which determines the ultimate cytokine profile. (**D**) 3. The composition of the Fc tail of the antibody, i.e., A; isotype, B; subclass, and C; glycosylation. (**E**) 4. The location and (local) concentration of antibody immune complexes. Natural barriers, such as epithelial cells mediate tolerance to bacteria, which is disrupted when epithelial layers are damaged, and IgG translocates to the lumen, while IgA translocates to the lamina propria. Abbreviations: TNF, tumor necrosis factor; IL, interleukin; TLR, Toll-like receptor; ADI, antibody-dependent inflammation; PRR, pattern recognition receptor.

Remarkably, while other antibody effector functions such as ADCP and ADCC are directly induced by FcRs, activation of FcRs alone does not induce substantial amounts of cytokines. In contrast, FcRs need to synergize with other receptors to modulate pro-inflammatory cytokine responses, such as the production of TNF, interleukins (IL) and interferons (IFNs) (Figure 2A). When invading pathogens are opsonized, they simultaneously activate FcRs and PRRs, leading to cross-talk that shapes the cytokine response to the pathogen involved.

For example, IgG opsonization of bacteria leads to FcγRIIa-TLR cross-talk that strongly and selectively promotes the amplification of cytokines such as IL-1β and IL-23, which promotes anti-bacterial Th17 responses [20,33]. In contrast, FcγRIIa-TLR cross-talk induced upon IgG opsonization of viruses strongly suppresses the production of type I and III IFNs, which is required to promote CD8 T cell responses [21]. This function of antibodies in controlling cytokine production emphasizes their role in promoting the collaboration between the adaptive and innate branch of our immune system by creating positive or negative feedback loops. Antibodies are formed by B cells (adaptive) upon recognition of antigens presented by APCs (innate), but only after proper T cell help (adaptive). Then, as part of their humoral function, antibodies opsonize pathogens and induce innate effector functions, but also induce cross-talk with PRRs and thereby promoting T cell skewing (adaptive), which is crucial for the induction of a strong adaptive immune response. These intricate feedback loops created by antibodies are important for shaping and continuous re-adjustment of host defense responses that are tailored to the situation at hand.

### 2.1. Molecular Mechanism of Antibody-Induced Inflammation

Because of its inflammatory potential, ADI is tightly controlled and critically depends on the fulfillment of four criteria: (1) the formation of immune complexes; (2) the availability of a co-stimulus (provided by other receptors such as TLRs); (3) the intrinsic properties of the antibody (isotype, subclass, and glycosylation); and (4) the location of the antibody-immune complexes (summarized in Figure 2). These four criteria act as “safety switches” for activation of ADI and will be summarized and discussed below.

#### 2.1.1. Complex Formation

A first requirement for ADI is the formation of immune complexes, i.e., the grouping of antibodies in close proximity, as occurs upon the binding of antibodies to their antigen. Antibodies are present in high concentrations in nearly all human tissues, with the exception of only a few compartments such as the central nervous system. Under homeostatic conditions, nearly all of these antibodies are present in a soluble, unbound form. Soluble antibodies elicit no immune response, or can even actively suppress inflammation [36,37]. Yet, the binding of antibodies to their antigen provides a “danger signal” by forming immune complexes, which is a prerequisite for ADI (Figure 2B). Interestingly, size seems to matter for the induction of ADI by antibody immune complexes. While several antibody effector functions such as phagocytosis are already induced by relatively small complexes, ADI requires immune complexes that are considerably larger [30,38,39]. However, the exact cutoff for the minimal immune complex size that is required for ADI has not yet been accurately determined. 

Our immune system is able to discriminate between unbound antibodies and immune complexes by differential activation FcRs. Binding of monomeric antibodies induce an inhibitory signaling pathway, known as inhibitory immunotyrosine-based activation motif signaling (ITAMi) [36,37]. However, large immune complexes activate other downstream signaling pathways. This switch mediated upon antibody-complex formation occurs not only for IgG [20,21,28,30], but can also occur for IgA [31,40].

The ability of FcRs to discriminate between unbound antibodies and immune complexes is of major importance to counteract infections with pathogens, because it provides a mechanism to trigger antibody effector functions specifically at the location that is infected (i.e., where microorganisms or infected cells are opsonized by antibodies), while no such immune responses are elicited in the rest of the body (despite very high systemic antibody concentrations).

#### 2.1.2. Co-Stimulation

Probably one of the reasons that ADI has long been underexposed compared to other antibody effector functions is that immune complex formation alone is generally insufficient, and an appropriate co-stimulus is indispensable. Most often, this co-stimulus is provided by the opsonized pathogen, which expresses PAMPs that activate various PRRs. Consequently, the combined stimulation of FcRs and PRRs results in FcR-PRR cross-talk [20,30,32,33], which is the driving force of ADI. A few exceptions to this requirement for a second stimulus have been described, in which antibody immune complexes under certain conditions can elicit a cytokine response by itself [41,42]. Interestingly these cytokine responses are induced by immune complexes that consist of antibodies that are described to be more pro-inflammatory, suggesting that some antibody-intrinsic differences can cause a pro-inflammatory cytokine response itself (we will elaborate on antibody-intrinsic differences in Section 2.1.3 and Section 3.1).

There are many different PRRs that can act as a co-stimulus to induce ADI, and the ultimate cytokine profile that is elicited upon FcR-PRR strongly depends on the PRR involved. For example, IgG opsonization of bacteria induces cross-talk of FcγRs with TLR2 or TLR4 (both bacteria sensing PRRs), which selectively upregulates the production of the pro-inflammatory cytokines IL-1β, IL-23, and TNF that promotes anti-bacterial Th17 responses [20,29,30,33]. In contrast, IgG opsonization of viruses or virus-infected cells induces cross-talk of FcγRs with virus sensing PRRs such as TLR3, which specifically downregulates the production of type I and III interferons (IFNs) that ultimately promotes anti-viral CD8^+^ T cell responses [21]. As such, ADI may play an important role in shaping pathogen class-specific immunity (Figure 2C).

While ADI induces bacteria and virus specific immune responses, it is currently still not completely clear whether antibody opsonization of fungi also promotes the activation of specific anti-fungal responses. Fungi (e.g., *Aspergillus*) can be recognized by various PRRs, including TLRs and CLRs, such as Dectin-1 [43,44]. While TLR stimulation can act as a co-stimulus for FcRs, little cross-talk was observed upon co-stimulation of FcγRs and Dectin-1 [30]. 

Although FcγR-TLR cross-talk upon both opsonized bacteria and viruses is regulated via the same receptor, i.e., FcγRIIa, distinct downstream signaling pathways are triggered, underlining the complexity of ADI. In contrast to pro-inflammatory cytokine induction upon FcyRIIa-TLR2 cross-talk, IFN suppression upon FcγRIIa-TLR3 cross-talk is Syk and PI3K independent [21,29]. The underlying molecular mechanism of the suppression of type I and III IFN upon cross-talk is not yet fully understood. The suppression of IFNs is not dependent on the production of pro-inflammatory cytokines such as TNF. Interestingly, FcγRIIa-TLR3 cross-talk suppresses the expression of interferon regulatory factor (IRF)1, a transcription factor for type I and II IFNs, but it is not yet clear whether this is fully responsible for the observed suppression [21]. 

Since for ADI the collaboration of FcRs with other receptors is essential, the key to understanding the molecular mechanism lies in the cross-talk between FcRs and its co-receptor. Recently, a crucial role was identified for the transcription factor IRF5 [29]. Initially, IRF5 was mainly considered to have a role in induction of IFNs, since it is able to bind to interferon stimulated response elements (ISRE) motifs [45]. IRF5 was later identified as an important marker to define inflammatory “M1” macrophages. IRF5 regulates transcription of pro-inflammatory cytokines in “M1” macrophages through binding to their promoter regions [46,47,48]. In addition, IRF5 polymorphisms are known to be associated with multiple chronic inflammatory disorders, including systemic lupus erythematosus (SLE), Sjögren Syndrome (SS), and rheumatoid arthritis (RA) [49,50,51,52].

**Figure 3:**
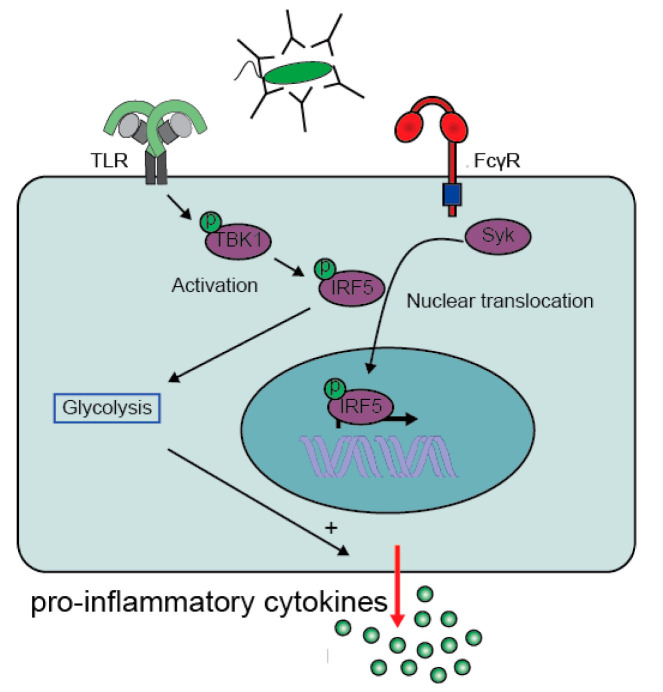
FcγR-TLR cross-talk converges on IRF5 activation. TLR activation mediates activation (phosphorylation) of IRF5 via activation of TBK1. Meanwhile, activation of FcγRIIa by IgG-immune complexes promotes translocation of the activated IRF5 to the nucleus. IRF5 is not only a transcription factor that increases gene transcription of pro-inflammatory genes, but also promotes glycolytic changes that enhances gene translation. Abbreviations: TBK1, TANK-binding kinase 1; IRF5, Interferon regulatory factor 5. (Modified figure from Hoepel et al. [29]).

More recently IRF5 was identified to play a central role in FcγR-TLR cross-talk, since both FcγR and TLR signaling pathways converge at IRF5 activation [29]. For full IRF5 activation, two steps are required, i.e., (1) phosphorylation and subsequent dimerization to activate IRF5, and (2) ubiquitination required for translocation of IRF5 to the nucleus [53]. IRF5 phosphorylation occurs after TLR2 signaling via the activation of the kinase TBK1 [29], while FcγR signaling is required to mediate IRF5 translocation [29]. Combined, the two receptors induce full IRF5 activation that ultimately promotes the production of pro-inflammatory cytokines by increasing both gene transcription and glycolysis (Figure 3) [29,54,55].

It is currently not fully understood which factor is required for the K63 ubiquitination and subsequent translocation of IRF5 [53,56]. The role of IRF5 in promoting inflammation is also clear from mouse models, where IRF5 promotes differentiation of Ly6Chi monocytes into CD11c^+^ macrophages, which increases the production of inflammatory mediators such as TNF, IL-1β, and IL-23 [57]. In humans not only TLR2, but also the intracellular TLR7, 8, and9 are able to phosphorylate IRF5, which is mediated by TLR adaptor interacting with SLC15A4 (TASL) that associates with SLC15A4. Upon association, TASL recruits and phosphorylates IRF5 via its conserved pLxIS [58].

#### 2.1.3. Antibody-Intrinsic Differences

Human antibodies exist in a broad variety. Beside the isotypes IgG, IgA, IgE, IgM, and IgD, immunoglobulins can be further subdivided based on subclasses and glycosylation (Figure 2D) [6,59]. These variations in antibodies affect their capacity to induce ADI, which is a tightly regulated and ingenious tool to mediate context-specific immunity. 

##### Isotype

Antibodies are abundantly present in most tissues of our body, yet the ratio between isotypes varies depending on the location. IgG is the most abundant isotype in human serum, while IgA is most ample in total, and particularly at mucosal sites. For several isotypes it has been identified that they mediate ADI. How antibodies steer the inflammatory responses upon FcR-PRR cross-talk is best established for IgG, both in the context of anti-bacterial [29] and anti-viral immunity [21]. However, IgA is similarly able to induce ADI by FcαRI-expressing cells. Although IgG is the most abundant isotype in human serum, blood also contains IgA that can amplify pro-inflammatory immune responses by monocytes and Kupffer cells, which may be important to counteract infections that enter the body via the vena porta [31]. At mucosal sites, IgA is by far the most abundant isotype. In the intestine, IgA can induce ADI by CD103^+^ DCs, a subset of intestinal DCs that are usually tolerogenic to bacteria, but whose tolerance is broken when they encounter IgA opsonized bacteria [40].

In contrast to IgG and IgA, IgE is bound to the FcεRI, and cross-linking and subsequent intracellular signaling occurs after binding of an antigen to the FcεRI-IgE composite [60]. Interestingly, FcεRI-mediated signaling in combination with TLR activation results in FcεRI-TLR cross-talk on basophils that induces the production of cytokines that promote the Th2 skewing of human naïve CD4^+^ T cells [61]. As such, this indicates the type of ADI that is elicited is dependent on the isotype involved.

While the FcRs for IgG, IgA, and IgE are well characterized and mainly expressed on innate immune cells [11,62], the expression pattern of the receptor for IgM (FcμR) is less clear, but seems to be mainly expressed by adaptive immune cells (e.g., B, T, and NK cells) [63,64]. It is tempting to speculate whether FcμR-PRR cross-talk would mediate an inflammatory response since some of these cells can also express PRRs, but this is thus far not described. In addition, the function of IgD is poorly understood, but it is described that it binds basophils and mast cells via a galectin and CD44 receptor complex [65,66]. Whether IgD-induced signaling induces cross-talk has thus far not been described.

##### Subclasses

Two out of the five antibody isotypes can be further subdivided into subclasses (Figure 2D). IgG and IgA comprise, respectively, four and two subclasses [6,59]. Importantly, different subclasses of the same isotype can mediate distinct inflammatory responses. This is illustrated by an experiment where *Streptococcus pneumoniae* bacteria were opsonized with the same antibody clone, but of different IgG subclasses [67]. While opsonization of bacteria with IgG1 and IgG2 amplified pro-inflammatory cytokine production, IgG3 did not. In contrast, IgG3 opsonization suppressed type I and III IFN production, while IgG2 immune complexes did not. These findings strongly suggest that IgG subclasses are involved in regulating pathogen-specific immunity, which would act on top of the pathogen-specific immune responses induced by the co-receptor.

Paradoxically, IgG3 is generally considered to be the most pro-inflammatory IgG subclass, which is particularly based on its short-half life and its capacity to strongly induce other antibody effector functions such as ADCC [5]. However, taking into account the capacity of IgG3 to induce cytokines, this reputation of IgG3 as the most inflammatory IgG subclass is likely to be an oversimplification. Instead, it appears that every different subclass has a different ability to induce the distinct antibody effector functions, such as ADCP, ADCC, complement activation, and ADI. As a result, there is a “division of labor” between IgG subclasses, with, e.g., IgG3, that is particularly efficient in inducing ADCC and complement activation, while IgG2 is particularly efficient in inducing ADI. IgG1 is very all-round by being able to activate nearly all antibody effector functions, while IgG4 is limited in inducing these effector functions (although it is more efficient in inducing ADI than IgG3) [67].

IgA consists of two subclasses, IgA1 and IgA2. For CD103^+^ DCs, FcαRI-TLR cross talk in humans with either IgA1 or IgA2 immune complexes did not show differences in the production of the pro-inflammatory cytokines TNF, IL-1β, and IL-23 [40]. In contrast, others showed that IgA2 isolated from human patients’ serum displays strong pro-inflammatory effects, while IgA1 does not. This effect was dependent on distinct glycosylation profiles between IgA1 and IgA2 [42]. An explanation for this difference in observations may be that Hansen et al. used monoclonal IgA subclasses, which are often myeloma-derived and are characterized by aberrant glycosylation profiles, while Steffen et al. used IgA antibodies isolated from healthy human serum [40,42,68]. 

##### Allotypes

Although multiple isotypes and subclasses are expressed in every single person, within an individual only one allotype is present per isotype. For IgG, 27 allotypes are known, of which IgG3 has the most different ones [69]. Interestingly, recent findings demonstrated that IgG3 allotypes showed large variation in its binding capacity to FcγRIIIa and subsequent induction of ADCC [69]. However, IgG allotypes show very little differences in the induction of ADI [67]. Yet, allotypes are still relevant from a holistic point of view, since in vivo antibodies execute multiple effector functions at the same time.

##### Glycosylation

In addition to antibody isotype and subclass, antibodies can also be differently glycosylated, which adds another layer to the diversity of ADI responses. Variations in glycosylation profile have functional consequences for their ability to induce effector functions [6,70,71,72]. There are multiple locations on antibodies that can be glycosylated, which affect their binding to FcRs, but may also affect the binding of antibodies to sugar-recognizing receptors such as lectins [6]. For IgG, a conserved N-linked glycan at position 297 in the constant Fc-domain has an important role for mediating various antibody effector functions, including ADI [6,70,73,74]. IgA bears multiple conserved sites for N-glycosylation, which varies amongst the IgA subclasses. IgA2 has four conserved N-regions, while IgA1 holds two. Interestingly, in addition to IgG and IgA2, IgA1 also bears several O-glycosylation sites in the hinge region (Figure 2D) [42,75].

While under homeostatic conditions most antibodies display similar antibody glycosylation patterns, the glycosylation of particular antibody clones can change under specific inflammatory conditions. For example, IgG glycosylation can be changed upon infection with enveloped viruses such as Dengue, HIV-1, or SARS-CoV-2 [70,76], which can have major effects on different antibody effector functions, including ADI [70,76].

#### 2.1.4. Location

On top of the three aforementioned criteria for ADI, there is a fourth requirement, which is the location of the antibody immune complexes. The importance of the location of antibodies for the induction of inflammation can be nicely illustrated by the compartmentalization of antibodies at mucosal surfaces, such as the airways or the intestine. Under homeostatic conditions, the main antibodies that are present in the lamina propria are unbound soluble IgA (dimers) and IgG (monomers). In contrast, the lumen is characterized by high amounts of (secretory) IgA that is actively transported in high amounts and forms immune complexes by binding to commensal bacteria, while the concentration of IgG in the lumen is restricted. 

When the mucosal layer is damaged, this homeostatic condition is disrupted. IgG, originating from the lamina propria can now opsonize bacteria originating from the lumen, which promotes formation of IgG immune complexes that induce FcγRIII-TLR cross-talk by activating epithelial cells on the luminal (apical) side [28]. Under homeostatic conditions, epithelial cells are unresponsive to Gram-negative bacteria, but in IgG immune complexes break this epithelial tolerance, leading to the production of pro-inflammatory cytokines and chemokines (Figure 2E) [28]. Simultaneously, IgA immune complexes, which under homeostatic conditions are only present in the lumen, will translocate to the lamina propria upon barrier damage, generating a strong pro-inflammatory immune response by immune cells (Figure 2E) [40] (reviewed in more detail in Hoepel et al. [77]). 

Translocation of antibodies may be of particular interest in the central nervous system, since it is one of the few organs where no antibodies are present under homeostatic conditions. Interestingly, microglia cells (macrophage-like cells in the CNS) are equipped with multiple FcγRs. While microglia are highly tolerogenic and hardly induce any inflammatory responses upon detection of microorganisms, stimulation with IgG immune complexes breaks the tolerance of microglia to microbial structures [35,78]. This indicates that the location of IgG immune complexes could serve a physiological purpose to (temporarily) break the tolerance in this immune-privileged tissue to counteract infections.

### 2.2. Inflammation by Pentraxins

ADI is induced through activation of FcRs. Interestingly, antibodies are not the only ligand for FcRs. FcRs can also be activated by proteins of the pentraxin family, which includes C-reactive protein (CRP), serum amyloid P component (SAP) and pentraxin member 3 (PTX3). From an evolutionary point of view, pentraxins are likely to be the original ligands of FcRs, since pentraxins occurred earlier in evolution than FcRs, while antibodies occurred substantially later [79]. Recent findings have demonstrated that pentraxins can induce inflammation reminiscent of ADI, although the molecular mechanisms appear to be different.

#### 2.2.1. Pentraxin Family 

Pentraxins are a family of phylogenetically highly conserved humoral fluid phase proteins, which are built of five identical subunits and are divided into short and long pentraxins based on the length of their primary structure [80]. The short pentraxins CRP and SAP are primarily produced in the liver as a response to pro-inflammatory cytokines. During infection their main function is to recognize a variety of pathogens and counteract their harmful effects by activation of the complement system as well as by binding to FcRs. In contrast, the long pentraxin PTX3 is rapidly produced by a variety of cells including macrophages, DCs, and endothelial cells during inflammatory conditions [80,81]. 

Pentraxins are phylogenetically highly conserved proteins. For example, CRP has been found in every organism, from the horseshoe crab to humans. Immunoglobulins on the other side are not that conserved and are highly diversified among species [82]. 

Pentraxin-induced inflammation has been most clearly described for CRP. CRP can recognize bacteria by binding to phosphocholine, which is a component of teichoic acid of Gram-positive bacteria, the lipopolysaccharides of Gram-negative bacteria, and microbial capsular carbohydrates [83]. Although CRP mainly binds bacteria, it also binds to parasites [84,85]. Interestingly, phosphocholine is also expressed by mammalian cells. However, this is not exposed on the outer membrane and CRP can only bind to phosphocholine when the cells are damaged or dying, which occurs upon apoptosis and particularly necrosis [79,86,87,88,89,90]. In addition to phosphocholine, CRP has been described to bind to other endogenous ligands that are exposed after cell damage or death, including ribonucleoproteins, chromatin, histones, and lysophosphatidylcholine [79,86,87,91,92]. Therefore, CRP-induced inflammation may not only play a role during pathogenic infections, but also during several inflammatory disorders, since there is often a lot of cell damage or death, which also results in the expression of DAMPs that can activated TLRs.

#### 2.2.2. Pentraxin-Induced Inflammation

When macrophages encounter CRP-opsonized bacteria, both PRRs and FcγRs will be stimulated simultaneously. Similar to IgG, bacterial opsonization with CRP results in a synergistic up-regulation of pro-inflammatory cytokines TNF, IL-1β, IL-6, and IL-23 by human PBMCs [82] and pro-inflammatory macrophages [93]. The modulated cytokine profile by FcR-TLR cross-talk skews Th cell responses towards Th17, which thereby tailors immune responses to counteract extracellular bacterial infections. The main FcRs responsible for the synergistic cytokine response are FcγRI and FcγRIIa [82,93]. 

**Figure 4:**
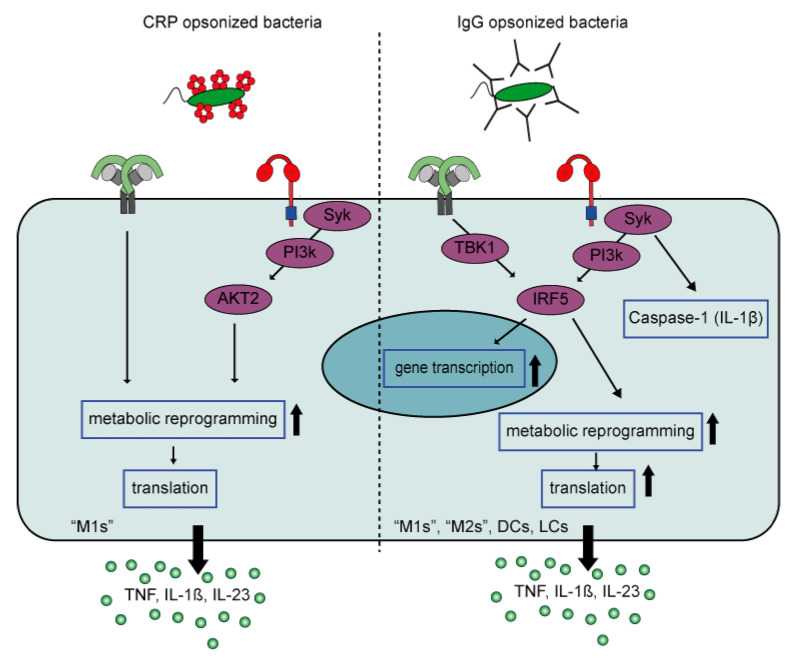
FcγR-TLR cross-talk of CRP-opsonized bacteria versus IgG-opsonized bacteria. FcγR-TLR cross-talk upon CRP opsonization of bacteria only amplifies cytokine production by M1 but not M2 macrophages, while IgG-opsonization of bacteria induces strong amplification by different myeloid cell types. In addition, CRP amplifies cytokine production by signaling though an AKT2 dependent pathway that induces metabolic reprogramming that only increases gene translation. In contrast, IgG signals through an IRF5 dependent pathway that amplifies cytokine production at three levels, i.e., gene transcription, gene translation, and caspase-1 activation (amplifying IL-1β production). Abbreviations; CRP, C-reactive Protein; M1, “pro-inflammatory” macrophages; M2, “anti-inflammatory” macrophages; DCs, dendritic cells; LCs, Langerhans cells.

Although CRP-induced inflammation by human pro-inflammatory macrophages is reminiscent of cytokine amplification by IgG immune complexes, there are several differences between CRP- and IgG-induced inflammation when looking at the underlying mechanism (Figure 4).

First, CRP appears to selectively amplify cytokine production induced by TLRs that signal through MyD88 (e.g., TLR2, 4, and 5), whereas IgG also amplifies cytokine production induced by TLR3, which signals through TRIF. This suggests that CRP particularly amplifies inflammation in response to (CRP-opsonized) bacteria, but not viruses. Second, while IgG-mediated amplification of pro-inflammatory cytokines is regulated at the level of (1) gene transcription, (2) gene translation, and (3) caspase-1 activation [67], the synergy between CRP and TLR ligands predominantly amplifies cytokine levels at the posttranscriptional level, by glycolytic reprogramming of inflammatory macrophages through signaling via kinases Syk, PI3K, and AKT2. Furthermore, in contrast to IgG, CRP does not amplify pro-inflammatory cytokine production by anti-inflammatory (“M2”) macrophages [93]. The reason why CRP only induces inflammation by pro-inflammatory (“M1”) macrophages, and not anti-inflammatory macrophages, may be explained by the metabolic differences between these cells. Recently, it has become clear that pro- and anti-inflammatory macrophages are metabolically different. While pro-inflammatory macrophages display a higher aerobic glycolysis (extracellular acidification rate; ECAR), as well a higher expression of genes encoding glycolytic enzymes, anti-inflammatory macrophages have a significantly higher oxygen consumption rate/ECAR ratio [94,95]. Another possibility may be the expression of IRF5, a downstream signalling molecule of FcγR-TLR cross-talk [29]. IRF5 is essential for pro-inflammatory macrophage polarization and IRF5 expression levels are substantially higher in pro-inflammatory macrophages compared to anti-inflammatory macrophages [48,96].

## 3. Pathological Immune Activation

**Figure 5:**
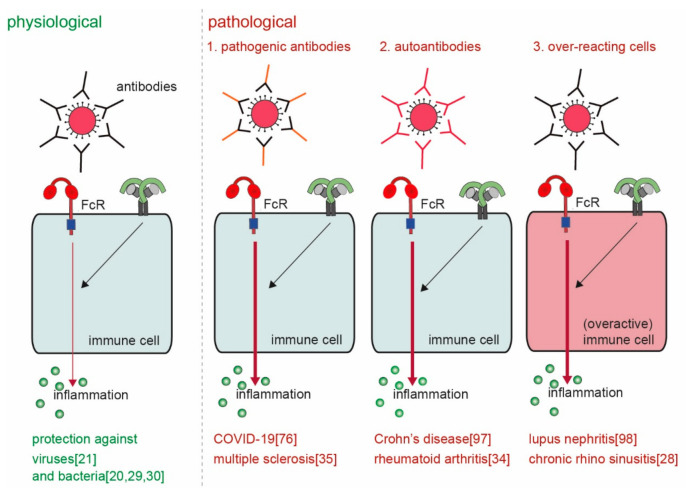
Physiological versus pathological ADI. ADI is a powerful physiological mechanism to counteract infections with invading pathogens such as bacteria and viruses [20,21,29,30]. However, pathological ADI can be cause by (a combination of) 1. pathogenic antibodies [35,76], 2. auto-antibodies [34,97], and 3. over-reacting cells [28,98].

The main physiological function of ADI is to provide host defense against invading pathogens. However, considered the very high levels of pro-inflammatory cytokines and chemokines that can be induced through this mechanism, undesired or aberrant activation can lead to severe pathology. ADI has been described to play a role in various diseases, including RA, SLE, inflammatory bowel disease (IBD), multiple sclerosis (MS), and recently also COVID-19. Although these diseases are very diverse in nature, the way by which ADI drives or promotes pathology from a mechanistic point of view can be categorized in four different groups (Figure 5).

First, antibodies can be more pro-inflammatory because of a different composition of the Fc tail. Second, ADI may be erroneously activated by antibodies that recognize self-structures (i.e., autoantibodies) instead of antibodies that recognize foreign structures. Third, cell-intrinsic differences in particular groups of individuals can lead to over-activation of ADI. Fourth, aberrant location (or increased concentrations) of antibody immune complexes can lead to undesired activation of ADI. These four different mechanisms of pathological ADI activation will be discussed below.

### 3.1. Pathogenic Antibodies

As discussed above, the composition of the Fc tail of antibodies has a major effect on the type of inflammatory response that is induced (e.g., anti-bacterial versus anti-viral), but also on the strength of the inflammatory response. Under particular circumstances, antibodies can be generated with an Fc tail composition that induces excessive inflammatory responses that lead to pathology (Figure 5). While all variables in Fc tail composition could be involved (i.e., isotype, subclass, allotype, and glycosylation), glycosylation appears to be most important regarding the induction of pathological inflammation by antibodies.

A key example of pathological ADI comes from recent studies on SARS-CoV-2, the virus that can cause COVID-19 [99,100]. While most people that are infected with SARS-CoV-2 only develop mild symptoms, some people develop severe and life-threatening disease [101]. Remarkably, these severely ill patients show a dramatic worsening of disease around the time of seroconversion, when antibodies against the virus are being produced [101]. Importantly, it has recently been identified that in severely ill patients, but not in patients with mild disease, the Fc tail of anti-spike IgG is aberrantly glycosylated [70]. Specifically, at position N297 in the Fc tail, these anti-spike antibodies display low amounts of fucose, and high amounts of galactose [70]. This change in glycosylation is known to increase the binding affinity to FcγRs, particularly FcγRIII [102]. Strikingly, this aberrant glycosylation of anti-spike dramatically amplifies pro-inflammatory cytokine production by alveolar macrophages, thereby contributing to the observed “cytokine storm” that is noticed in these patients [76]. Moreover, this macrophage (over)activation subsequently permeabilizes pulmonary endothelium, leading to flooding of the lungs, and induces microvascular thrombosis, two hallmarks of severe COVID-19 [76,103]. Previously, similar effects had already been observed upon infection with SARS-CoV-1 [104]. Since many enveloped viruses can induce IgG with low fucose and high galactose [70], this pathological form of ADI may also occur upon other severe infections with other (non-corona) viruses.

In addition to viral infections, aberrant glycosylation of antibodies has been described for several other disorders. These include a number of autoimmune diseases, such as RA [105,106] and MS [107]. In RA, these antibodies may promote inflammation through activation of synovial macrophages [32,34]. Interestingly, in RA not only IgG, but also IgA shows intrinsic differences that promote inflammation, both regarding glycosylation and by a shift from the IgA1 subclass to the more pro-inflammatory IgA2 [42]. In MS patients, IgG antibodies in the cerebrospinal fluid show a clearly different glycosylation profile compared to in blood of these patients [107]. Although it has not yet been tested whether these glycosylation changes promote inflammation in the CNS, recent findings do demonstrate that the majority of MS patients has brain-bound IgG, and that these IgG immune complexes elicit pro-inflammatory cytokine production by primary human microglia [35].

### 3.2. Autoantibodies

A second way by which ADI can lead to pathology, is when antibodies are not made in response to foreign antigens, but instead to self-structures (Figure 5). This occurs in autoimmune diseases, where autoantibodies bind to self-antigens and form immune complexes that undesirably activate macrophages to promote inflammation. Inflammation by autoantibodies has been described for various autoimmune diseases, including SLE [108] and RA [109]. In RA, autoantibodies are mostly directed against citrullinated proteins [110,111], while in SLE autoantibodies are mostly directed against nuclear structures such DNA and RNA [108,112]. However, as described earlier, IgG immune complex formation alone is not sufficient for a strong induction of pro-inflammatory cytokines, and a “second signal” is required to promote inflammation. In the joints of RA patients, this second signal most likely comes from DAMPs that arise upon tissue damage, and which can activate TLRs [34]. In SLE patients, the second signal is most likely provided by self-DNA and self-RNA that stimulates endosomal TLRs [113,114,115]. While RA and SLE are key examples, ADI by autoantibodies is likely to also occur for various other autoimmune diseases, such as systemic sclerosis (SSc), SS, pemphigus, and MS [115,116,117,118,119,120,121]. 

### 3.3. Cell-Intrinsic Overactivation

A third way by which ADI can lead to over-activation, is by cell-intrinsic differences in immune cells of particular individuals (Figure 5). For example, nearly all SLE patients have IgG autoantibodies directed against nuclear antigens that form immune complexes. However, it was identified that immune cells form SLE patients that suffer from kidney inflammation (i.e., lupus nephritis) produce far more pro-inflammatory cytokines and type I IFN in response to IgG immune complexes than SLE patients without nephritis [98]. This was not dependent on differences in medication [98] and is most likely caused by genetic or epigenetic cell-intrinsic differences in this subset of patients. A second example comes from patients that suffer from chronic rhinosinusitis with nasal polyps (CRSwNP). While healthy nasal epithelium is immune tolerant for commensal Gram-negative bacteria, this tolerance is broken when a breach in the epithelial layer leads to FcγR stimulation on the apical side of the epithelial cells (Figure 2E). However, in nasal epithelium of CRSwNP patients, this mechanism is constitutively activated [28], leading to continuous inflammatory responses to commensal bacteria.

In addition, cell-intrinsic differences could play a role for various other autoimmune diseases. The main receptor inducing ADI in RA and SLE is FcγRIIa [34,112], which signals through the transcription factor IRF5 (Figure 4) [29]. Interestingly, polymorphisms in the *IRF5* gene are associated with disease severity in many chronic inflammatory disorders, including SLE, SS, IBD, and RA [45,52,57,122,123,124,125]. This suggests that genetic risk factors may contribute to ADI-driven pathology.

### 3.4. Aberrant Location/Concentration

Finally, the location and/or local concentration of antibody immune complexes could lead to over-activation of ADI. For example, under homeostatic conditions no antibodies are present in the central nervous system. However, in pathological conditions such as MS, oligoclonal IgG is present in the central nervous system, which forms IgG immune complexes by binding to structures such as myelin that subsequently breaks the otherwise tolerogenic phenotype of microglia [35]. While this inflammatory response is usually transient after infection with pathogens, oligoclonal IgG is produced for extensive periods of time in MS patients, thereby perpetuating chronic inflammation. Alternatively, IgG and/or IgA expression can be locally increased, and thereby potentiate tissue-specific inflammatory responses. This has been described for both forms of IBD, i.e., Crohn’s disease (CD) and ulcerative colitis (UC). In UC, increased levels of IgG are found against commensal microorganisms, leading to increased presence of IgG immune complexes that (over)activate intestinal macrophages [97]. In CD, in has recently been identified that NOD2 deficiency increases the local presence of IgA immune complexes in the lamina propria of the intestine, thereby promoting intestinal inflammation and dysbiosis [126].

In addition to antibodies, also increased and/or prolonged concentrations of CRP may promote chronic inflammation. Although the soluble levels of acute-phase protein CRP can be very high upon various infections and inflammatory conditions, these will not directly lead to inflammation. Yet, when CRP forms complexes by binding to either particular bacterial strains or cell debris, this complexed CRP strongly promotes pro-inflammatory cytokine production by macrophages. For example, complexed CRP activates foamy macrophages that are formed in atherosclerotic plaques [93]. Since CRP binds to oxidized Low-density lipoprotein (LDL) and dead cells in the cholesterol-rich necrotic core in atherosclerotic plaques [127,128], and elevated CRP levels are one of the main risk factors for cardiovascular diseases [129], CRP-induced inflammation may contribute to the pathology in atherosclerosis. Similarly, CRP levels are increased in the inflamed joints of RA patients [130], where they are likely to form complexes because of local tissue damage, and thereby could promote the production of RA-associated cytokines such as TNF. CRP could also play a role in severely ill COVID-19 patients, since CRP levels in these patients are exceptionally high [70] and severe tissue damage in the lungs will lead to ample binding of CRP. In these patients, this would then act on top of the inflammation that is already induced by pathogenic IgG [76].

Finally, it is important to realize that these four mechanisms of pathological ADI activation could also be occurring simultaneously. For example, autoimmune diseases such as RA are not only characterized by autoantibodies but can also express antibodies with aberrant glycosylation and/or elevated CRP levels. The same could occur for diseases such as IBD (increased local IgG and CRP concentrations, and potential cell-intrinsic over-activation by polymorphisms in FcγRIIa and/or IRF5) and SLE (autoantibodies, and cell-intrinsic over-activation in SLE patients with nephritis). These combinations may add up to increase the risk and/or severity of the different diseases, thereby underling the potential relevance of aberrant macrophage activation in these diseases.

## 4. Therapeutic Opportunities

As described above, erroneous activation of ADI can have dramatic consequences, such as persistent infection, chronic inflammation, and autoimmunity. Unraveling the mechanisms behind ADI will not only increase our understanding of the pathogenesis of several disorders but may also yield new potential therapeutic options. In this final part, we will discuss the potential therapeutic implications of ADI-induced physiology and pathophysiology.

### 4.1. Inducing Controlled ADI to Boost Host Defense

Since ADI is a powerful mechanism to promote inflammatory responses against pathogens, it may be a helpful tool to induce adequate immune responses against pathogens that cause long-lasting or recurrent infections, such as *Mycobacterium tuberculosis* and *Staphylococcus aureus* [131]. This could be accomplished by generating antibodies with particular inflammatory features regarding isotype, subclass, and glycosylation. However, for these antibodies it will be very important to closely monitor the safety, since over-activation (as, e.g., observed in COVID-19) may cause extensive collateral damage.

Another approach to apply our new insights on ADI is for the development of new vaccines. Vaccines are a long established strategy to provide humoral immunity against a broad range of pathogens [132]. Nevertheless, for many pathogens, including HIV and *Plasmodium* (malaria), no sufficient vaccine is currently available [133,134]. An essential part of a vaccine is a solid adjuvant to boost the immune response. By using specifically designed antibodies as an adjuvant, the cytokine response and subsequent skewing of the immune response can be directed towards, e.g., Th17 or CD8^+^ T cell activation [20,21], thereby increasing the efficacy of the vaccine.

Finally, the knowledge surrounding ADI could be applied for the development of antibody-based therapies. In the last decade, antibody-based therapies have become an indispensable part of our treatment strategies, which are used to treat various diseases, e.g., by blocking of receptors or neutralization of cytokines [135]. Remarkably, the overwhelming majority of these antibodies are of the IgG1 subclass [59,135]. The reasons are its predominant presence in human serum, the all-round effector functions, and the fact that the IgG1 backbone was the first to be approved for use as therapeutic antibodies [59]. The use of other subclasses may provide a more tailored approach to steer towards either an anti-bacterial, anti-viral, or immune suppressive response. Particularly in combination with tailored glycosylation patterns, this may enable very specific fine-tuning of the desired immune response. Yet, this does require more elaborate knowledge on the effect of glycosylation of different subclasses.

### 4.2. Interfering with Uncontrolled ADI in Chronic Inflammation

While ADI provides a powerful mechanism to counteract infections, over-activation of this mechanism can lead to chronic inflammation. One example of how ADI contributes to chronic inflammation is when antibodies become intrinsically more pro-inflammatory, caused by changes in the antibody glycosylation as described for various diseases, including COVID-19 [70,76], RA [42,106], and multiple myeloma [136]. Therefore, targeting of antibody glycosylation may be a promising therapeutic option. Interestingly, pre-clinical data already showed that oral administration of N-acetylmannosamine can enhance antibody-sialylation and thereby reduce its pro-inflammatory capacity [106,136]. Another example of over-activation of ADI is provided by UC, one of the two main forms of IBD [137]. UC is characterized by higher amounts of IgG directed against commensal bacteria, and this isotype switch is associated with increased ADI and subsequent chronic inflammation [97]. To counteract this uncontrolled activation of ADI, targeting FcγR signaling would be an interesting approach. Yet, this requires in depth knowledge of the mechanisms that underlies FcR-induced inflammation. While single nucleotide polymorphisms (SNPs) in FcRs that change their affinities for antibodies are associated with diseases such as CD and UC, there are conflicting findings on whether these SNPs have a direct effect on antibody-induced inflammation [30,97,138]. This discrepancy may be related to the involvement of other FcR-dependent functions that are affected by these SNPs (e.g., phagocytosis and complement activation), but could also be related to a phenomenon known as epistasis, meaning that the effect of the FcR SNPs may be dependent on other genes [139]. Based on findings by us and others, IRF5 poses as an interesting therapeutic target [29,140,141,142,143,144,145]. However, we should be careful with inhibiting IRF5 completely. Although reducing IRF5 attenuates UC, it also impairs the clearance of intestinal pathogens [146], underlining its crucial role in balancing host defense and chronic inflammation. Since IRF5 has different cellular functions that seem to be controlled by different post-translational modifications (including phosphorylation and K63-ubiquitination) [147], an important future strategy may be not tosimply reduce IRF5 expression altogether, but to specifically target the pathways upstream of IRF5 that skew mucosal immunity towards (chronic) inflammation. Recent findings on antibody-induced IRF5 activation by both classical FcRs and TRIM21 [148] provide numerous targets for therapy that could be blocked using small molecule inhibitors that inhibit kinases, E3 ligases, or metabolic processes.

Another interesting molecule to target in chronic inflammation is Syk. Fostamatinib, which inhibits Syk, is a drug recently approved by the EMA and FDA [149]. Fostamatinib is being tested in a phase II clinical trial in high-risk IgA nephropathy [150,151]. More recently, the active compound of fostamatinib was used to abolish inflammation elicited by inflammatory antibodies of severely ill COVID-19 patients [76]. It would be interesting to investigate this drug (as well as other drugs that target this kinase) in other FcR-induced inflammatory diseases.

Undesired activation of ADI occurs when antibodies do not recognize microorganisms, but self-structures, which leads to pathology in autoimmune diseases such as SLE [108] and RA [109,152]. Interestingly, for many of these autoimmune diseases, polymorphisms in the *IRF5* gene are associated with disease severity, including SLE, SS, and RA [45,52,122,123,124]. Therefore, targeting IRF5 may not only be an interesting approach to treat chronic inflammation, but also for autoimmune diseases. Although it is still unclear whether MS can be categorized as a bona fide autoimmune disease [78,116], it was found that IgG immune complexes are bound to myelin of the majority of MS patients [35]. In addition, IRF5 polymorphisms are also associated with MS [153,154]. Given the presence of IRF5 in microglia cells [155], inhibition of IRF5 in human microglia cells may also be used as a strategy to prevent excessive inflammation in MS.

## 5. Concluding Remarks

Antibodies and pentraxins can induce physiological and pathological inflammation. Both the physiological and pathological function of ADI may provide new therapeutic options to treat diseases. However, when designing antibodies to evoke ADI, or when designing drugs that counteract ADI, it is important to realize that ADI is just one of many antibody effector functions, and other processes such as phagocytosis and ADCC may also be affected simultaneously. Another complication is that ADI seems to be particularly pronounced in humans, but is restricted in species that are generally used for in vivo experiments, such as mice [22]. Better in vivo models would be valuable to not only assess the in vivo effect of ADI, but also to determine how it is intertwined with the other antibody effector functions. By continuing these efforts to unravel the mechanisms behind ADI, we may not only be able to find new ways to counteract chronic inflammation, but also to improve the growing field of antibody therapy.

## Data Availability

No applicable.
